# Supplementary material for: Dynalign II: common secondary structure prediction for RNA homologs with domain insertions
Source: Nucleic Acids Res. 2014 Nov 21;42(22):13939–48. doi: 10.1093/nar/gku1172 (PMC4267632; doi:10.1093/nar/gku1172)
Supplement: SUPPLEMENTARY DATA [file supp_gku1172_nar-02021-z-2014-File012.zip › manual/RNA_class/html/class_turbo_fold.html]

RNAstructure Classes: TurboFold Class Reference

- Main Page
- Classes

- Class List
- Class Hierarchy
- Class Members

# TurboFold Class Reference

TurboFold Class.
More...

`#include <TurboFold_object.h>`

List of all members.

|  |  |
| --- | --- |
|  |  |
| Public Member Functions | |
|  | TurboFold (const char fasta\_fp[]) |
|  | Constructor - user provides a filename for a FASTA file. |
|  | TurboFold (vector< string > \*sequences, vector< string > \*saves) |
|  | Constructor - user provides a vector array of strings that provide input and output file names. |
|  | ~TurboFold () |
|  | Destructor. |
| int | GetErrorCode () |
|  | Get an integer that reports the current error status of the class. |
| char \* | GetErrorMessage (int err\_code) |
|  | Return error messages based on code from GetErrorCode or function-returned error codes. |
| string | GetErrorString (int err\_code) |
|  | Return error messages based on code from GetErrorCode and other error codes. |
| int | SetMaxPairingDistance (int distance) |
|  | Set a maximum distance between nucleotides that can pair. |
| int | ReadSHAPE (const int i\_seq, const char fp[], const double par1, const double par2) |
|  | Read and apply SHAPE mapping data to a specific sequence. |
| int | SetTemperature (double temp) |
|  | Set the folding temperature. |
| int | fold (double gamma=0.3, int n\_iterations=3, int \_n\_parallel\_pfunctions=1) |
|  | The main TurboFold algorithm. |
| int | ProbKnot (const int i\_seq, const int n\_iterations, const int minhelixlength) |
|  | Use the ProbKnot algorithm to predict a structure for a sequence. |
| int | PredictProbablePairs (const int i\_seq, const float probability) |
|  | Predict a structure for a sequence that is composed of highly probably pairs. |
| int | MaximizeExpectedAccuracy (const int i\_seq, const double maxPercent, const int maxStructures, const int window, const double gamma=1.0) |
|  | Predict maximum expected accuracy structures for a sequence. |
| int | GetPair (const int i\_seq, const int i, const int structurenumber=1) |
|  | Provide pairing information. |
| double | GetPairProbability (const int i\_seq, const int i, const int j) |
|  | Provide pairing probability information. |
| int | GetNumberSequences () |
|  | Provide the number of sequences used in the calculation. |
| int | WriteCt (const int i\_seq, const char fp[]) |
|  | Write the predicted structures for a specific sequence to a ct file. |
| void | SetProgress (TProgressDialog &Progress) |
| void | StopProgress () |

---

## Detailed Description

TurboFold Class.

The TurboFold class provides an entry point for the TurboFold algorithm in RNAstructure. 

---

## Constructor & Destructor Documentation

|  |  |  |  |  |  |
| --- | --- | --- | --- | --- | --- |
| TurboFold::TurboFold | ( | const char | *fasta\_fp*[] | ) |  |

Constructor - user provides a filename for a FASTA file.

Input file should contain two or more FASTA sequences. The constructor reads parameter files from disk. The location should be specified in the DATAPATH environment variable. If DATAPATH is undefined, the program will attempt to load the files from the present working directory. This constructor generates internal error codes that can be accessed by GetErrorCode() after the constructor is called. 0 = no error. The errorcode can be resolved to a c string using GetErrorMessage.

**Parameters:**
:   |  |  |  |
    | --- | --- | --- |
    |  | *fasta\_fp* | is a NULL terminated c string that give a filename. This must be 1000 or fewer characters. |

|  |  |  |  |  |
| --- | --- | --- | --- | --- |
| TurboFold::TurboFold | ( | vector< string > \* | *sequences*, |  |
|  |  | vector< string > \* | *saves* |  |
|  | ) |  |  |  |

Constructor - user provides a vector array of strings that provide input and output file names.

The output files are partition function save files, which can be read by the RNA class to determine ppair probabilities. There needs to be one output file per input sequence. The constructor reads parameter files from disk. The location should be specified in the DATAPATH environment variable. If DATAPATH is undefined, the program will attempt to load the files from the present working directory. This constructor generates internal error codes that can be accessed by GetErrorCode() after the constructor is called. 0 = no error. The errorcode can be resolved to a c string using GetErrorMessage.

**Parameters:**
:   |  |  |  |
    | --- | --- | --- |
    |  | *sequences* | is a vector of strings that provide sequence file names. These files need to be either FASTA or .seq. |
    |  | *saves* | is a vector of strings that provide file names for output partition function save files. This array needs to have exactly the same number of elements as sequences. |

|  |  |  |  |  |
| --- | --- | --- | --- | --- |
| TurboFold::~TurboFold | ( |  | ) |  |

Destructor.

this->aln\_mapping\_probs[i\_seq1][i\_seq2][i] = (double\*)malloc(sizeof(double) \* (max\_k - min\_k + 2));

---

## Member Function Documentation

|  |  |  |  |  |
| --- | --- | --- | --- | --- |
| int TurboFold::fold | ( | double | *gamma* = `0.3`, |  |
|  |  | int | *n\_iterations* = `3`, |  |
|  |  | int | *\_n\_parallel\_pfunctions* = `1` |  |
|  | ) |  |  |  |

The main TurboFold algorithm.

This function accomplishes the task of determining the pair probabilities. This function must be called before any of the structure prediction methods can be used.

**Parameters:**
:   |  |  |  |
    | --- | --- | --- |
    |  | *gamma* | is the weight of the extrinsic information. Larger gamma will result in more consistent structures. The default is 0.3 and this provided a good structure prediction accuracy in benchmarks. |
    |  | *n\_iterations* | is the number of iterations that should be performed to converge the base pairing probabilities. The default is 3 because benchmarks showed only marginal improvement with further iterations. |
    |  | *\_n\_parallel\_pfunctions* | is the number of threads to use. For code compiled in serial, this must be 1, which is the default. Define COMPILE\_SMP to build for multithreading. |

**Returns:**
:   An integer error code that can be resolved to an error message using GetErrorMessage() or GetErrorString(). 0 is no error.

|  |  |  |  |  |
| --- | --- | --- | --- | --- |
| int TurboFold::GetErrorCode | ( |  | ) |  |

Get an integer that reports the current error status of the class.

Functions generate internal errors that can be accessed using this function. An error code of zero is no error. A non-zero error code can be resolved to a cstring or string using GetErrorMessage() or GetErrorString().

**Returns:**
:   An integer that indicates error status.

|  |  |  |  |  |  |
| --- | --- | --- | --- | --- | --- |
| char \* TurboFold::GetErrorMessage | ( | int | *err\_code* | ) |  |

Return error messages based on code from GetErrorCode or function-returned error codes.

**Parameters:**
:   |  |  |  |
    | --- | --- | --- |
    |  | *err\_code* | is the integer error code provided by GetErrorCode(). |

**Returns:**
:   A pointer to a c string that provides an error message.

|  |  |  |  |  |  |
| --- | --- | --- | --- | --- | --- |
| string TurboFold::GetErrorString | ( | int | *err\_code* | ) |  |

Return error messages based on code from GetErrorCode and other error codes.

**Parameters:**
:   |  |  |  |
    | --- | --- | --- |
    |  | *err\_code* | is the integer error code provided by GetErrorCode() or from other functions that return integer error codes. |

**Returns:**
:   A string that provides an error message.

|  |  |  |  |  |
| --- | --- | --- | --- | --- |
| int TurboFold::GetNumberSequences | ( |  | ) |  |

Provide the number of sequences used in the calculation.

**Returns:**
:   The number of sequences used in the calculation.

|  |  |  |  |  |
| --- | --- | --- | --- | --- |
| int TurboFold::GetPair | ( | const int | *i\_seq*, |  |
|  |  | const int | *i*, |  |
|  |  | const int | *structurenumber* = `1` |  |
|  | ) |  |  |  |

Provide pairing information.

This function can only be called after one of the structure prediction methods is a called. This function generates internal error codes that can be accessed by GetErrorCode() after the constructor is called. 0 = no error. The errorcode can be resolved to a c string using GetErrorMessage.

**Parameters:**
:   |  |  |  |
    | --- | --- | --- |
    |  | *i\_seq* | is the sequence number, where the number starts at 1. |
    |  | *i* | is the nucleotide. |
    |  | *structurenumber* | is the structure number. This can be used to specify a suboptimal structure, but defaults to 1. |

**Returns:**
:   The nucleotide to which i is paired in sequence i\_seq and suboptimal structure number structurenumber. Zero indicates that the nucleotide is unpaired.

|  |  |  |  |  |
| --- | --- | --- | --- | --- |
| double TurboFold::GetPairProbability | ( | const int | *i\_seq*, |  |
|  |  | const int | *i*, |  |
|  |  | const int | *j* |  |
|  | ) |  |  |  |

Provide pairing probability information.

This function can only be called after fold() is a called. This function generates internal error codes that can be accessed by GetErrorCode() after the constructor is called. 0 = no error. The errorcode can be resolved to a c string using GetErrorMessage.

**Parameters:**
:   |  |  |  |
    | --- | --- | --- |
    |  | *i\_seq* | is the sequence number, where the number starts at 1. |
    |  | *i* | is the 5' nucleotide in a pair. |
    |  | *j* | is the 3' nucleotide in a pair. |

**Returns:**
:   The probability that i is paired to j in sequence i\_seq.

|  |  |  |  |  |
| --- | --- | --- | --- | --- |
| int TurboFold::MaximizeExpectedAccuracy | ( | const int | *i\_seq*, |  |
|  |  | const double | *maxPercent*, |  |
|  |  | const int | *maxStructures*, |  |
|  |  | const int | *window*, |  |
|  |  | const double | *gamma* = `1.0` |  |
|  | ) |  |  |  |

Predict maximum expected accuracy structures for a sequence.

This function can only be called after fold() is called. The expectd accuracy score for a structure is = gamma \* 2 \* (sum of pairing probabilities for pairs) + (sum of unpairing probabilities for single stranded nucleotides).

**Parameters:**
:   |  |  |  |
    | --- | --- | --- |
    |  | *i\_seq* | is the sequence number, where the number starts at 1. |
    |  | *maxPercent* | is the maximum difference in score allowed for generation of suboptimal structures. |
    |  | *maxStructures* | is the maximum number of suboptimal structures allowed. |
    |  | *window* | is the window parameter that controls what suboptimal structures can be included. 0 is the minimum and the higher the window, the more different suboptimal structures must be from each other. |
    |  | *gamma* | is the weight on base pairs. The default of 1.0 works well based on benchmarks on single sequence calculations. |

**Returns:**
:   An integer error code that can be resolved to an error message using GetErrorMessage() or GetErrorString(). 0 is no error.

|  |  |  |  |  |
| --- | --- | --- | --- | --- |
| int TurboFold::PredictProbablePairs | ( | const int | *i\_seq*, |  |
|  |  | const float | *probability* |  |
|  | ) |  |  |  |

Predict a structure for a sequence that is composed of highly probably pairs.

This function can only be called after fold() is called.

**Parameters:**
:   |  |  |  |
    | --- | --- | --- |
    |  | *i\_seq* | is the sequence number, where the number starts at 1. |
    |  | *probability* | is the pairing probability threshold, where pairs will be predicted if they have a higher probability. Note that a value of less than 0.5 (50%), will cause an error. The default value of zero will trigger the creation of 8 structures, with thresholds of >=0.99, >=0.97, >=0.95, >=0.90, >=0.80, >=0.70, >=0.60, >0.50. |

**Returns:**
:   An integer error code that can be resolved to an error message using GetErrorMessage() or GetErrorString(). 0 is no error.

|  |  |  |  |  |
| --- | --- | --- | --- | --- |
| int TurboFold::ProbKnot | ( | const int | *i\_seq*, |  |
|  |  | const int | *n\_iterations*, |  |
|  |  | const int | *minhelixlength* |  |
|  | ) |  |  |  |

Use the ProbKnot algorithm to predict a structure for a sequence.

This function can predict pseudoknots. This function can only be called after fold() is called.

**Parameters:**
:   |  |  |  |
    | --- | --- | --- |
    |  | *i\_seq* | is the sequence number, where the number starts at 1. |
    |  | *n\_iterations* | is the number of ProbKnot iterations. |
    |  | *minhelixlength* | is the length of the shortest helix allowed. |

**Returns:**
:   An integer error code that can be resolved to an error message using GetErrorMessage() or GetErrorString(). 0 is no error.

|  |  |  |  |  |
| --- | --- | --- | --- | --- |
| int TurboFold::ReadSHAPE | ( | const int | *i\_seq*, |  |
|  |  | const char | *fp*[], |  |
|  |  | const double | *par1*, |  |
|  |  | const double | *par2* |  |
|  | ) |  |  |  |

Read and apply SHAPE mapping data to a specific sequence.

The pseudofree energy approach will be used to apply SHAPE data to restrain structure prediction. Where DG(per stack with ith nucleotide) = slope (SHAPE on ith nucleotide) + intercept. This function must be called before fold().

**Parameters:**
:   |  |  |  |
    | --- | --- | --- |
    |  | *i\_seq* | is the sequence number to which the restraint should be applied, where the number starts at 1. |
    |  | *fp[]* | is a cstring that provides the name of the file that contains the normalized SHAPE mapping data. |
    |  | *par1* | is the slope in kcal/mol. |
    |  | *par2* | is the intercept in kcal/mol. |

**Returns:**
:   An integer error code that can be resolved to an error message using GetErrorMessage() or GetErrorString(). 0 is no error.

|  |  |  |  |  |  |
| --- | --- | --- | --- | --- | --- |
| int TurboFold::SetMaxPairingDistance | ( | int | *distance* | ) |  |

Set a maximum distance between nucleotides that can pair.

This function must be called before fold() and will limit the distance between nucleotides that can pair.

**Parameters:**
:   |  |  |  |
    | --- | --- | --- |
    |  | *distance* | is an integer that specifies the maximum distance between nucleotides that can pair, , i.e. \|j-i\| < distance for nucleotide i to pair to j. |

**Returns:**
:   An integer error code that can be resolved to an error message using GetErrorMessage() or GetErrorString(). 0 is no error.

|  |  |  |  |  |  |
| --- | --- | --- | --- | --- | --- |
| void TurboFold::SetProgress | ( | TProgressDialog & | *Progress* | ) |  |

Provide a TProgressDialog for following calculation progress. A TProgressDialog class has a public function void update(int percent) that indicates the progress of a long calculation.

**Parameters:**
:   |  |  |  |
    | --- | --- | --- |
    |  | *Progress* | is a TProgressDialog class. |

|  |  |  |  |  |  |
| --- | --- | --- | --- | --- | --- |
| int TurboFold::SetTemperature | ( | double | *temp* | ) |  |

Set the folding temperature.

This function must be called before fold(). If this function is not called, the default temperature of 310.15 K (37 degrees C) is used.

**Parameters:**
:   |  |  |  |
    | --- | --- | --- |
    |  | *temp* | is the temperature in Kelvin. |

**Returns:**
:   An integer error code that can be resolved to an error message using GetErrorMessage() or GetErrorString(). 0 is no error.

|  |  |  |  |  |
| --- | --- | --- | --- | --- |
| void TurboFold::StopProgress | ( |  | ) |  |

Provide a means to stop using a TProgressDialog. StopProgress tells the RNA class to no longer follow progress. This should be called if the TProgressDialog is deleted, so that this class does not make reference to it.

|  |  |  |  |  |
| --- | --- | --- | --- | --- |
| int TurboFold::WriteCt | ( | const int | *i\_seq*, |  |
|  |  | const char | *fp*[] |  |
|  | ) |  |  |  |

Write the predicted structures for a specific sequence to a ct file.

This function can only be called after one of the structure prediction methods is a called.

**Parameters:**
:   |  |  |  |
    | --- | --- | --- |
    |  | *i\_seq* | is the sequence number, where the number starts at 1. |
    |  | *fp* | is a cstring that gives the filename to which the ct table is to be written. |

**Returns:**
:   An integer error code that can be resolved to an error message using GetErrorMessage() or GetErrorString(). 0 is no error.

---

The documentation for this class was generated from the following files:

- E:/RNAstructure/TurboFold/TurboFold\_object.h- E:/RNAstructure/TurboFold/TurboFold\_object.cpp

---

Generated on Wed Sep 7 12:53:49 2011 for RNAstructure Classes by 
 1.5.7.1
